# Genetic Variability and Population Structure of the Mushroom *Pleurotus eryngii* var. *tuoliensis*

**DOI:** 10.1371/journal.pone.0083253

**Published:** 2013-12-12

**Authors:** Mengran Zhao, Chenyang Huang, Qiang Chen, Xiangli Wu, Jibin Qu, Jinxia Zhang

**Affiliations:** Key Laboratory of Microbial Resources, Ministry of Agriculture, Institute of Agricultural Resources and Regional Planning, Chinese Academy of Agricultural Sciences, Beijing, P.R. China; Smithsonian Conservation Biology Institute, United States of America

## Abstract

The genetic diversity of 123 wild strains of *Pleurotus eryngii* var. *tuoliensis*, which were collected from nine geographical locations in Yumin, Tuoli, and Qinghe counties in the Xinjiang Autonomous Region of China, was analysed using two molecular marker systems (inter-simple sequence repeat and start codon targeted). At the variety level, the percentage of polymorphic loci and Nei’s gene diversity index for *P. eryngii* var. *tuoliensis* was 96.32% and 0.238, respectively. At the population level, Nei’s gene diversity index ranged from 0.149 to 0.218 with an average of 0.186, and Shannon's information index ranged from 0.213 to 0.339 with an average of 0.284. These results revealed the abundant genetic variability in the wild resources of *P. eryngii* var. *tuoliensis*. Nei’s gene diversity analysis indicated that the genetic variance was mainly found within individual geographical populations, and the analysis of molecular variance revealed low but significant genetic differentiation among local and regional populations. The limited gene flow (Nm = 1.794) was inferred as a major reason for the extent of genetic differentiation of *P. eryngii* var. *tuoliensis*. The results of Mantel tests showed that the genetic distance among geographical populations of *P. eryngii* var. *tuoliensis* was positively correlated with the geographical distance and the longitudinal distances (r_Go_ = 0.789 and r_Ln_ = 0.873, respectively), which indicates that geographical isolation is an important factor for the observed genetic differentiation. Nine geographical populations of *P. eryngii* var. *tuoliensis* were divided into three groups according to their geographical origins, which revealed that the genetic diversity was closely related to the geographical distribution of this wild fungus.

## Introduction

The *Pleurotus* mushroom, which is commercially called Bai Ling Gu, is a precious edible fungus of high nutrient and medicinal value [1,2]. In the wild, this mushroom parasitically or saprophytically grows together with *Ferula* plants in the Umbelliferae family [3]. It has a restricted habitat in the northwest part of China, i.e., Xinjiang Autonomous Region [4]. According to the traditional criteria of fungal classification, taxonomic status of this wild fungus is not clear yet [4-6]. The results of rDNA sequence analysis, mating, and cultivation tests indicated that the *Pleurotus* mushroom found specifically in China is a branch of *Pleurotus eryngii*, which evolved independently in China [7]. The scientific name was then defined as *Pleurotus eryngii* var. *tuoliensis* through morphological identification and internal transcribed spacer (ITS) analysis [8].

*P. eryngii* species complex has the most abundant population diversity in the genus *Pleurotus*. It consists of at least six varieties [9]. Because it is important both economically and ecologically, this species complex has attracted more and more attention worldwide [10-13]. Analyses via molecular markers revealed a high level of heterogeneity within *P. eryngii* var. *eryngii*, var. *ferulae*, and var. *nebrodensis* [14]. The genetic diversity of *P. eryngii* var. *eryngii* and var. *ferulae* was more abundant than that of var. *nebrodensis*, var. *Elaeoselinum*, and var. *Thapsia* [15]. The analysis of the population structure of *P. eryngii* var. *eryngii* and var. *ferulae* revealed a greater variation within geographical populations than that between geographical populations [16].

Inter-simple sequence repeats (ISSR) markers have been widely applied to analyses of genetic variance and population structure in many types of organisms [17-21]. Because a molecular marker system usually targets to detect a particular genome region, the level of genetic diversity might not be objectively revealed using a single marker system. Previous studies have shown that ISSR analyses only showed a relatively low genetic diversity among peanut cultivars despite abundant morphological, physiological, and agronomic variance [22]. Nevertheless, a molecular marker system termed start codon targeted (SCoT) polymorphism, which is a simple and novel DNA marker system, could detect more polymorphisms compared with several other molecular marker systems [23,24]. The SCoT marker system was employed not only in the study of genetic relationships but also in revealing the geographical origins of cultivars [24-28]. Xiong et al. [29] suggested that the SCoT marker system could be used as an effective supplement to ISSR and RAPD. Additional genetic variances can be reflected through the comprehensive use of various molecular marker systems [30,31].

In this study, the genetic variation of 123 samples of *P. eryngii* var. *tuoliensis* was studied using ISSR and SCoT markers. The goals of this study were to investigate the genetic structure characteristic and the level of genetic variation of *P. eryngii* var. *tuoliensis*, and to thereby infer the factors that influence the genetic differentiation in the populations.

## Materials and Methods

### Ethics Statement

Although the wild mushroom of *P. eryngii* var. *tuoliensis* is not considered an endangered species, commercial collecting nowadays is forbidden for protecting the local ecological environment, but collecting for research purposes is allowed. Anyone who collects the wild mushroom of *P. eryngii* var. *tuoliensis* for commercial purpose will be fined by the local Agricultural Bureaus (Agricultural Bureaus of Yumin County, Agricultural Bureaus of Tuoli County, and Agricultural Bureaus Qinghe County of Xinjiang Autonomous Region of China).

### Sampling

**Figure 1:**
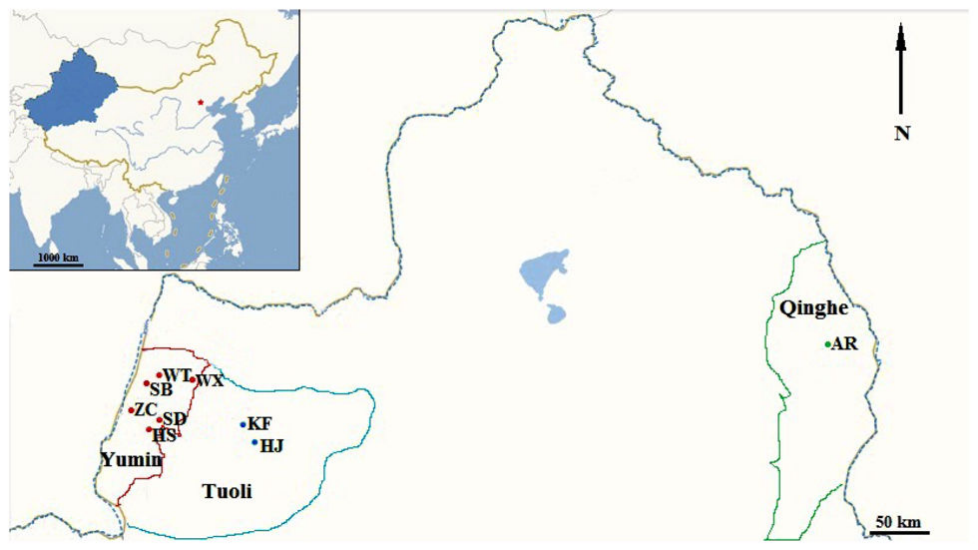
Sampling sites of *Pleurotus eryngii* var. *tuoliensis* in China. The Xinjiang Autonomous Region is highlighted in blue in the map of China shown in the upper left. WX, ZC, SB, SD, HS, and WT in the Yumin region are marked as red rotundities; KF and HJ in the Tuoli region are marked as blue rotundities; and AR in the Qinghe region is marked as a green rotundity.

Based on the natural occurrence of fruiting bodies, we confirmed the number of sampling sites in Yumin, Tuoli, and Qinghe. A total of 123 wild samples were collected from nine sites in three regions of Xinjiang from March to May of 2009 (Figure 1). These sampling regions spanned approximately 650 km from the east to the west and approximately 150 km from the south to the north. The sample size and the geographical coordinates for each geographical population are presented in Table 1.

**Table 1:** Geographical and climatic data of the P. *eryngii* var. *tuoliensis* populations.

Geographic Region	Annual Temperature (°C ) *	Annual Rainfall (mm) *	Geographic Population	Sample Size	Latitude (North)	Longitude (East)
Yumin	7.7	291	WX	16	46.35	83.31
			ZC	24	46.10	82.84
			SB	9	46.28	82.90
			SD	8	45.98	82.99
			HS	17	45.85	82.94
			WT	32	46.45	82.96
Tuoli	6.0	245	KF	6	45.95	83.72
			HJ	3	45.85	83.86
Qinghe	1.3	189	AR	8	46.50	90.34
			Total samples	123		

^*^ , The values were the average of 30 years (1981-2010). Data from: http://www.cma.gov.cn/2011qxfw/2011qsjgx/

### Sample isolation

Pure culture of each strain was obtained by isolating tissue culture from the fruiting bodies. A small piece of tissue was removed aseptically and transferred into a culture tube containing potato dextrose agar (PDA), and incubated in the dark at 25°C for 7-9 days. These samples were next stored at 4°C until needed.

### DNA extract

Mycelia for DNA extraction were cultured on PDA Petri dishes with cellophane at 25°C for 7 days. The total DNA was extracted using a DP305-Plant Genome Extraction Kit (Tiangen, China). The purity and quality of the genomic DNA were determined through spectrophotometry and electrophoresis on 1.0% agarose gel. The DNA solution was stored at -20°C.

### ISSR analysis

Seven ISSR primers were selected in a pre-experiment (Table 2). The amplification reactions were performed in a final volume of 20 μL containing 20 ng of the template DNA, 10X Ex *Taq* buffer, 0.2 mM dNTPs, 0.5 mM of each primer, and 1 U of Ex *Taq* DNA polymerase (TaKaRa). The PCR reaction conditions were the following: initial denaturation step at 94°C for 4 min, 35 cycles of 50 s at 94°C, annealing at 55°C for 50 s, and extension at 72°C for 2 min, and final extension at 72°C for 7 min. The amplified products were resolved on a 1.0% agarose gel and stained with ethidium bromide. The results were observed through an ultraviolet gel imaging system. The molecular weights of DNA bands on the agarose gel were estimated using a DNA ladder, i.e., 2-log (TaKaRa).

**Table 2:** ISSR and SCoT primer sequences for *P. eryngii* var. *tuoliensis*.

Molecular Marker	Primer	Sequence (5'→3')
ISSR	P1	TGCACACACACACAC
	P2	GTGACACACACACAC
	P4	GGATGCAACACACACACAC
	P10	GAGAGAGAGAGAGAGAC
	P19	ACACACACACACACACCT
	P24	CACGAGAGAGAGAGAGA
	P864	ATGATGATGATGATGATG
SCoT	S13	ACGACATGGCGACCATCG
	S14	ACGACATGGCGACCACGC
	S19	ACCATGGCTACCACCGGC
	S27	ACCATGGCTACCACCGTG
	S28	CCATGGCTACCACCGCCA
	S29	CCATGGCTACCACCGGCC
	S30	CCATGGCTACCACCGGCG
	S31	CCATGGCTACCACCGCCT

### SCoT analysis

Eight out of 36 SCoT primers were selected, which produced clear and reproducible profiles [23]. Sequences of the SCoT primer are listed in Table 2. The PCR mix consisted of a total volume of 20 μL containing 2 μL of 10X Ex *Taq* buffer, 0.25 mM dNTPs, 0.5 mM of each primer, 1 U of Ex *Taq* DNA polymerase (TaKaRa), and 20 ng of the template DNA. The PCR reaction was performed using the following thermal cycling protocol: 94°C for 5 min, 35 cycles of 94°C for 1 min, 55°C for 1 min, and 72°C for 2 min. The reaction was completed through incubation at 72°C for 10 min. The SCoT products were resolved through electrophoresis on 1.5% agarose gels.

### Data analysis

The gels were scored for the presence or absence of reproducible bands. Each band was regarded as a locus with two alternative alleles. The data from the ISSR and the SCoT analyses were converted into a binary data matrix as discrete variables (1 = presence and 0 = absence). To expand the range of DNA polymorphisms assayed in a genome and to increase the reliability of the results, the diversity analyses were performed in this study using combined data of ISSR and SCoT.

The genetic variability at the variety or population level was analysed under the assumption of Hardy-Weinberg equilibrium. The percentage of polymorphic bands (PPB), the numbers of effective alleles (Ne), Shannon’s information index (I), and Nei’s gene diversity index (H) were calculated using the GenAIEx 6.41 software [32].

According to the method developed by Nei, the gene diversity statistics, including the total allelic diversity (Ht), the mean allelic diversity within populations (Hs), the proportion of the total allelic diversity found among populations (Gst), and the gene flow among populations (Nm), were obtained using the POPGENE (v 1.31) [33]. The total genetic variation among the samples was calculated using the phi-statistic through the analysis of molecular variance (AMOVA). This analysis was performed using the computer program GENALEX [32]. The total genetic variation is partitioned at three levels—within populations (Phi-PT), among populations within regions (Phi-PR), and among regional populations (Phi-RT) [34].

The pairwise Nei’s genetic distances between populations were calculated using the POPGENE software. In order to examine whether local populations that are geographically close are genetically similar, correlation of genetic distances with geographical, latitudinal and longitudinal distances respectively was analyzed through three Mantel tests using the GENALEX program [32].

The cluster analysis for the populations was performed with the NTSYSpc-2.1e program [35] based on the Nei’s genetic distances using the UPGMA (Unweighted Pair Group Method with Arithmetic Mean). 

## Results

### Genetic variation at the variety and population level

The analysis of the genetic diversity among 123 samples of *P. eryngii* var. *tuoliensis* showed that the percentage of polymorphic bands, the number of effective alleles, Nei’s gene diversity index, and Shannon’s information index were 96.32%, 1.381, 0.238, and 0.377, respectively. The results from these analyses suggested that there are high levels of genetic variations within this variety. 

The percentage of polymorphic bands among the nine geographical populations ranged from a low of 33.44% for the HJ population in Tuoli to a high of 77.59% for the WT population in Yumin with an average of 57.75%. In addition, Nei’s gene diversity index ranged from a minimum of 0.149 to a maximum of 0.218 with an average of 0.186, whereas Shannon’s Information index ranged from 0.213 to 0.339 with an average of 0.284 (Table 3). The two parameters were highest for the ZC population in Yumin and lowest for the HJ population in Tuoli.

**Table 3:** Analysis of the genetic variation of *P. eryngii* var. *tuoliensis*.

Population	Percentage of Polymorphic Loci	No. of Effective Alleles	Shannon’s Information Index	Gene Diversity
WX	60.54%	1.303 (0.020)	0.284 (0.015)	0.184 (0.11)
ZC	75.59%	1.355 (0.020)	0339 (0.014)	0.218 (0.010)
SB	51.17%	1.289 (0.020)	0.263 (0.016)	0.174 (0.011)
SD	53.18%	1.312 (0.021)	0.280 (0.016)	0.186 (0.011)
HS	69.90%	1.326 (0.019)	0.316 (0.015)	0.203 (0.010)
WT	77.59%	1.332 (0.019)	0.324 (0.014)	0.206 (0.010)
KF	45.48%	1.301 (0.021)	0.259 (0.017)	0.175 (0.012)
HJ	33.44%	1.268 (0.022)	0.213 (0.017)	0.149 (0.012)
AR	52.84%	1.303 (0.020)	0.276 (0.016)	0.182 (0.011)
Population level	57.75%	1.310 (0.007)	0.284 (0.005)	0.186 (0.004)
Species level	96.32%	1.381 (0.019)	0.377 (0.012)	0.238 (0.009)

The values in parentheses brace denote the standard errors.

### Genetic differentiation between populations and levels of gene flow

The results from Nei’s genetic diversity analysis showed that the average total genetic diversity (Ht) over all loci was 0.206 for *P. eryngii* var. *tuoliensi*s and that the genetic diversity within populations (Hs) was 0.161. The relative degree of gene differentiation among the nine populations (Gst) was 0.218. In other words, 21.8% and 78.2% of the genetic diversity was between populations and within populations, respectively, which reveals that a large amount of the genetic diversity was found within populations.

The results of AMOVA suggested significant genetic differences within populations. The results obtained from the Nei’s genetic diversity analysis were consistent. Specifically, 13% of the total genetic variance (PhiRT) was found between populations from different regions. The next level, i.e., among populations within regions, contributed 8% of the total genetic variance (PhiPR), and the remaining 79% genetic variance was obtained from within individual populations (PhiPT). All of the three levels contributed significantly to the overall genetic variation, as determined through the permutation analyses (Table 4).

**Table 4:** Summary of the AMOVA results for 123 specimens of *P. eryngii* var. *tuoliensis*.

Source	d.f.	SS	MS	Estimated Variance	Percentage %	Phi Statistic	Value	*P*
Among Regions	2	272.85	136.43	5.24	13%	PhiRT	0.130	0.01
Among Populations within Regions	6	481.74	80.29	3.29	8%	PhiPR	0.094	0.01
Within Populations	114	3622.32	31.78	31.78	79%	PhiPT	0.212	0.01
Total	122	4376.91		40.31	100%			

d.f., degree of freedom; SS, sum of squared observations; MS, mean of squared observations; PhiRT, proportion of the total genetic variance that is due to the variance between regions; PhiPR, proportion of the total genetic variance that is due to the variance among populations within a region; PhiPT, proportion of the total genetic variance that is due to the variance among individuals within a variant.

The gene flow (Nm) estimate obtained by Gst was 1.794 for *P. eryngii* var. *tuoliensis*. Compared with those of other basidiomycetes [36,37], the level of gene flow that was present between nine populations was relatively low. 

### Relationship between genetic distance and geographical distribution

**Figure 2:**
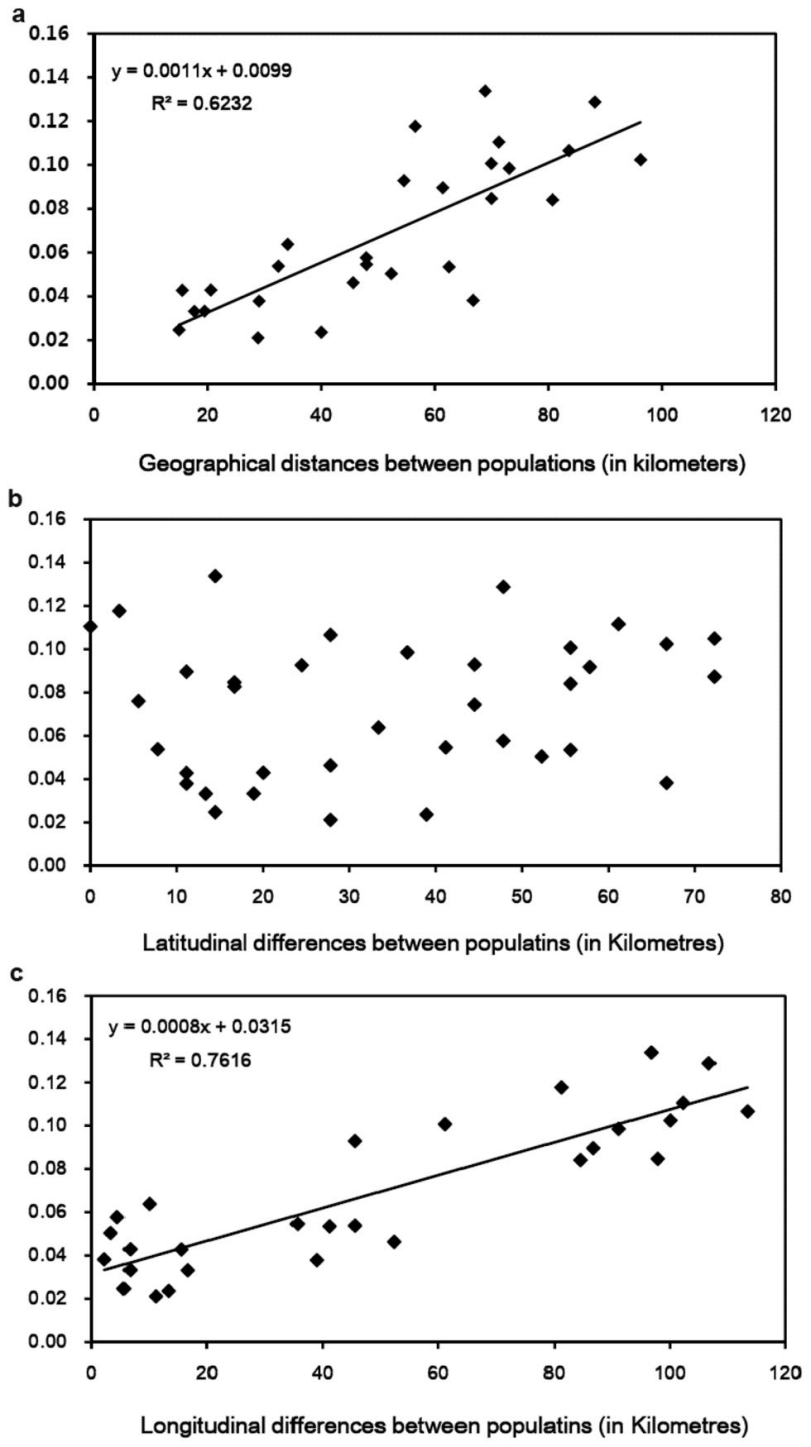
Mantel tests between the genetic distance and the geographical parameters. (a) Mantel test between Nei’s genetic distance and the geographical distance among populations. (b) Mantel test between Nei’s genetic distance and the latitudinal distance. (c) Mantel test between Nei’s genetic distance and the longitudinal distance. In 2a, 2b, and 2c, the X-axis represents the geographical parameter, and the Y-axis represents Nei’s genetic distances between the populations.

The genetic distances between populations of *P. eryngii* var. *tuoliensis* varied from 0.021 to 0.134 (Table 5). The smallest genetic distance was observed between the ZC population and the HS population, both of which are located in the same region (Yumin). The largest genetic distance was found between the SD population of Yumin and the HJ population of Tuoli. The results showed that the genetic distance between the populations located in the same region was smaller than that obtained between populations from different regions. The results from the Mantel tests showed a significant positive correlation between genetic distance and geographical distance among all of the tested populations except for the AR population, which is located approximately 500 km from the other 8 populations (Figure 2a; r_Go_ = 0.789, *P* = 0.02). Moreover, we found that the longitudinal differences, rather than the latitudinal differences, were associated with the genetic distance (Figure 2b; r_Lt_ = 0.157, *P* = 0.24; Figure 2c; r_Ln_ = 0.873, *P* = 0.01).

**Table 5:** Pairwise Nei’s genetic distances between geographical populations of *P. eryngii* var. *tuoliensis*.

	WX	ZC	SB	SD	HS	WT	KF	HJ
ZC	0.046							
SB	0.054	0.043						
SD	0.055	0.033	0.064					
HS	0.054	0.021	0.058	0.025				
WT	0.038	0.024	0.033	0.050	0.038			
KF	0.093	0.085	0.099	0.118	0.090	0.084		
HJ	0.101	0.107	0.129	0.134	0.111	0.102	0.043	
AR	0.083	0.074	0.093	0.092	0.087	0.076	0.112	0.105

### Cluster analysis

**Figure 3:**
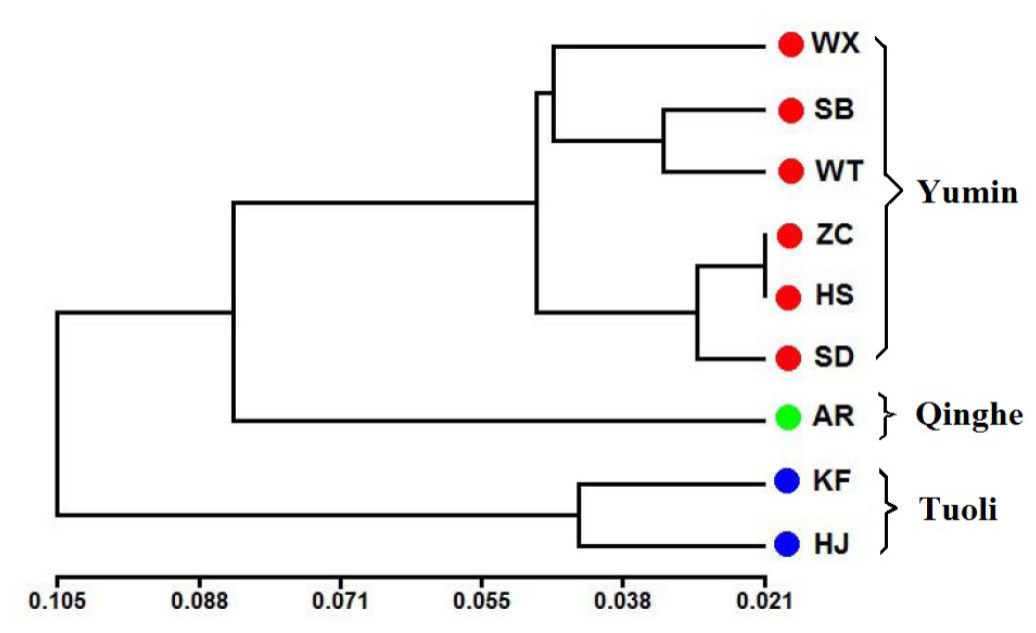
UPGMA dendrogram of the nine populations of *P. eryngii* var. *tuoliensis* based on Nei’s genetic distance. The scale bar means the genetic distance.

Nine geographical populations of *P. eryngii* var. *tuoliensis* were divided into three groups according to their geographical origins, which indicate that the genetic diversity is closely related to the geographical distribution. The populations from six locations in Yumin were group together in the first cluster, and the second cluster consisted of the two populations from Tuoli. The AR population located in Qinghe was first clustered with the Yumin group, whereas the Tuoli group was far from the above two groups and clustered last (Figure 3).

## Discussion

Two dominant molecular markers (ISSR and SCoT) were used to evaluate the genetic diversity of the wild mushroom of *P. eryngii* var. *tuoliensis* in the study. Compared to results obtained with co-dominant markers, this type of markers usually underestimate the within-population variation while produce similar or somewhat higher population differentiation parameters [38-40]. These differences were expected since dominant markers can only produce two alleles in each locus, while they cannot distinguish between homozygotes and heterozygotes [41]. Despite these disadvantages, dominant markers are characterized by estimating unbiased genetic variation and no sequence requirement, which makes them appropriate to analyze those species of little or no genetic information [39,42].

In fungi, high levels of genetic variability are usually observed in wild populations that reproduce sexually, have broad ecological niches, and/or have a wide geographical distribution [43]. The percentage of polymorphic bands (PPB) and Nei’s gene diversity index (H) are two important parameters that are used to measure the genetic variation at the species level [44]. Our results showed a particularly high level of genetic polymorphism. Similar polymorphism levels were previously observed in other edible mushrooms, such as *Lentinula edodes* (99.6%) [45] and *Auricularia polytricha* (99.8%) [46]. The mushroom of *P. eryngii* var. *tuoliensis*, which independently evolved in China, is a major branch of the P. *eryngii* species complex and is only distributed in the north area of Xinjiang. The geographical distribution of this variety was not as wide as that observed for *P. eryngii* var. *eryngii* or var. *ferulae* [47]. In general, widespread species were more diverse compared to stenochoric species [48]. However, our analyses indicated that the level of genetic variation of *P. eryngii* var. *tuoliensis* is higher than those of the other varieties (PPB = 84.6% in var. *ferulae*; PPB = 38.5% in var. *nebrodensis*, var. *Elaeoselinum*, and var. *Thapsia*) in the species complex, with the exception of *P. eryngii* var. *eryngii* [15]. This result may be mainly attributed to the special climate of Xinjiang, which exhibits dry and windy climate conditions and obvious temperature variations. The intraspecies genetic diversity was closely related to the horizontal transmission system of propagules (airborne spores) and to the adaptation to stressful and temporally heterogeneous environments [13]. The dry and windy climate in Xinjiang contributed to the spore dispersal. Moreover, the Spearman rank correlation revealed that the gene diversity exhibited a strong positive association with the aridity index and the temperature [11]. Stressful (e.g., dry and high temperature) and temporally heterogeneous environments tend to increase the genetic diversity in basidiomycetes of higher classification status [49,50].

The genetic variance was partitioned within populations rather than among populations of *P. eryngii* var. *tuoliensis* (Gst = 0.218), and this result is similar to the findings obtained previously in several basidiomycete species. For example, the intercontinental populations of the model basidiomycete *Schizophyllum commune* were differentiated significantly with a Gst value of 0.214 [43]. For the wood-decay fungus *Phlebia centrifuga*, eight populations across northern Europe showed a Gst value of 0.072 [51]. The Gst value for geographical populations of *Tricholoma matsutake* respectively from southwestern, and northeastern China was found to be approximately 0.10 [34,52]. The natural populations of heterothallic basidiomycetes are composed of individual secondary mycelia, which are somatically incompatible and genetically and physiologically distinct. The population structure was regulated through the somatic compatibility systems and mating systems. Some studies have led to the generalization that outcrossing species maintain the majority of the genetic variability within populations, whereas inbreeding species hold most variability among populations [43].

Efficient patterns of dispersal, establishment and spread of the fungi appear to be other factors that influence the local genetic structure of populations. Fungal populations are established by two different dispersal mechanisms. The structure of local populations that are mainly maintained through basidiospore dispersal is characterised by a large number of genetically different individuals within a bit substrate, as has been observed with *Pleurotus ostreatus* [53] and *Mycena rosea* [54]. The populations are maintained not by direct basidiospore dispersal but through rapid vegetative spread in the leaf litter or soil. These populations often contain a few but widespread genotypes or just one genotype, as was found for *Armillaria bulbosa* [55] and *Lycoperdon pyriforme* [56], respectively. The partitioning pattern of the genetic variation of *P. eryngii* var. *tuoliensis*, which corresponds to those of the Italian populations of *P. eryngii* and *P. ferulae*, as shown by the fact that most of the variations are contained within local populations [16], would indicate that basidiospore dispersal is the main dissemination mechanism for populations of *P. eryngii* var. *tuoliensis*. 

Our results found significant genetic differentiation between populations of *P. eryngii* var. *tuoliensis* (Table 4). This finding is inconsistent with the results obtained from a previous study about *P. eryngii* and *P. ferulae* [16]. It was inferred that the degree of differentiation between populations could be affected by the level of gene flow. In some sense, the population structure was coordinated through two forces: gene flow and genetic drift. A higher level of gene flow would lead to a more homogeneous population structure and a lower extent of genetic differentiation. The gene flow within populations of *P*. *eryngii* var. *tuoliensis* was obviously lower than those of *P. eryngii* and *P. ferulae*, which exhibit Nm values of 3.50 and 5.43, respectively [16]. The level of gene flow might not be strong enough to counter the local genetic differentiation between certain populations, although in theory, the differentiation caused by genetic drift could be prevented when the number of migrants per generation (Nm) exceeds one [57].

The results from the Mantel tests and the cluster analysis supported the hypothesis that the genetic relationship among populations was closely associated with their geographic distributions (Tables 2 and 3). Compared with the latitudinal differences, the longitudinal differences played a more important role in the local genetic differentiation between populations. This observation is in agreement with the results obtained by Lewinsohn et al. [58] for the P. *eryngii* species complex in Israel. Although geographically Yumin and Tuoli distribute 73 km apart and share similar climates, the degree of population divergence between these two regions was higher than that obtained between Qinghe and the other two regions, which are approximately 500 km away from Qinghe. This unexpected finding might be attributed to the peculiar geographical positions of Yumin and Tuoli. Yumin and Tuoli lie in the northwestern Xinjiang region, which is prone to strong winds. During the fruiting season of *P*. *eryngii* var. *tuoliensis* (March to May), the northwest winds prevail in these regions. The dry windy climate might contribute to the long-distance transmission of spores. The gene flow mediated by spore dispersal might weaken the effect of the geographical distance isolation to some extent. The microclimate in Yumin is similar to that in Tuoli, but the populations within these two regions are effectively separated by hills. Studies of biogeography have indicated that certain particular ecological factors can cause population divergence at the microscale. Natural geographical barriers, including mountains and/or rivers, are one of the most important factors [59,60].
